# Emulsion Liquid Membranes Based on Os–NP/n–Decanol or n–Dodecanol Nanodispersions for p–Nitrophenol Reduction

**DOI:** 10.3390/molecules29081842

**Published:** 2024-04-18

**Authors:** Andreia Pîrțac, Aurelia Cristina Nechifor, Szidonia-Katalin Tanczos, Ovidiu Cristian Oprea, Alexandra Raluca Grosu, Cristian Matei, Vlad-Alexandru Grosu, Bogdan Ștefan Vasile, Paul Constantin Albu, Gheorghe Nechifor

**Affiliations:** 1 Analytical Chemistry and Environmental Engineering Department, University POLITEHNICA of Bucharest, 1-7 Polizu St., 011061 Bucharest, Romania; andreia.pascu@yahoo.ro (A.P.); aureliacristinanechifor@gmail.com (A.C.N.); andra.grosu@upb.ro (A.R.G.); 2 Department of Bioengineering, University Sapientia of Miercurea-Ciuc, 500104 Miercurea-Ciuc, Romania; tczszidonia@yahoo.com; 3 National Research Center for Micro and Nanomaterials, University POLITEHNICA of Bucharest, 060042 Bucharest, Romania; ovidiu.oprea@upb.ro (O.C.O.); vasile_bogdan_stefan@yahoo.com (B.Ș.V.); 4 National Research Center for Food Safety, University POLITEHNICA of Bucharest, Splaiul Independentei 313, 060042 Bucharest, Romania; 5 Faculty of Chemical Engineering and Biotechnologies, University POLITEHNICA of Bucharest, 1-7 Polizu St., 011061 Bucharest, Romania; 6 Academy of Romanian Scientists, Ilfov Street 3, 050044 Bucharest, Romania; 7 Department of Inorganic Chemistry, Physical Chemistry and Electrochemistry, University POLITEHNICA of Bucharest, 1-7 Polizu St., 011061 Bucharest, Romania; cristian.matei@upb.ro; 8 Department of Electronic Technology and Reliability, Faculty of Electronics, Telecommunications and Information Technology, University POLITEHNICA of Bucharest, 061071 Bucharest, Romania; 9 Radioisotopes and Radiation Metrology Department (DRMR), IFIN Horia Hulubei, 023465 Măgurele, Romania; paulalbu@gmail.com

**Keywords:** osmium, osmium nanoparticle, osmium reduction, p–nitrophenol reduction, nanodispersion, liquid membranes, emulsion liquid membranes, undecylenic acid, n–dodecanol, n–decanol

## Abstract

Membrane materials with osmium nanoparticles have been recently reported for bulk membranes and supported composite membrane systems. In the present paper, a catalytic material based on osmium dispersed in n–decanol (nD) or n–dodecanol (nDD) is presented, which also works as an emulsion membrane. The hydrogenation of p–nitrophenol (PNP) is carried out in a reaction and separation column in which an emulsion in the acid-receiving phase is dispersed in an osmium nanodispersion in n–alcohols. The variables of the PNP conversion process and p–aminophenol (PAP) transport are as follows: the nature of the membrane alcohol, the flow regime, the pH difference between the source and receiving phases and the number of operating cycles. The conversion results are in all cases better for nD than nDD. The counter-current flow regime is superior to the co-current flow. Increasing the pH difference between the source and receiving phases amplifies the process. The number of operating cycles is limited to five, after which the regeneration of the membrane dispersion is required. The apparent catalytic rate constant (*k_app_*) of the new catalytic material based on the emulsion membrane with the nanodispersion of osmium nanoparticles (0.1 × 10^−3^ s^−1^ for n–dodecanol and 0.9 × 10^−3^ s^−1^ for n–decanol) is lower by an order of magnitude compared to those based on adsorption on catalysts from the platinum metal group. The advantage of the tested membrane catalytic material is that it extracts p–aminophenol in the acid-receiving phase.

## 1. Introduction

**Figure 1:**
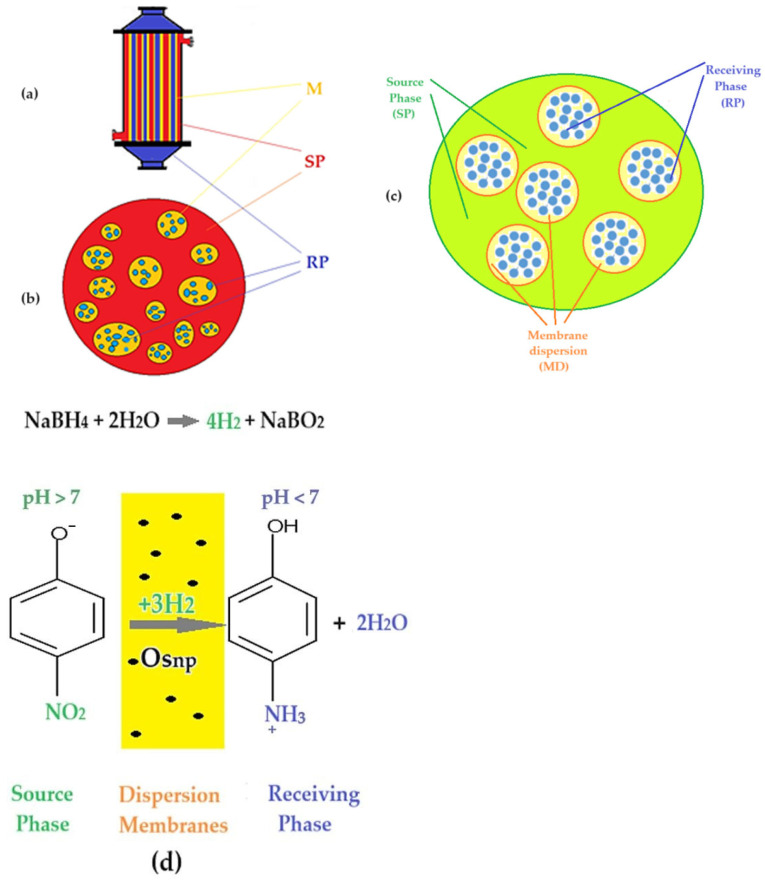
(**a**) Supported liquid membrane (SLM); (**b**) emulsion liquid membrane (ELM); (**c**) proposal reactional membrane systems; (**d**) catalytic p–nitrophenol reduction with molecular hydrogen through osmium–nanoparticle membrane [19,20,30,31]; M: membrane (Os–NP/n–alcohol dispersion); SP: source phase (p–nitrophenol + sodium tetrahydride in alkaline aqueous solution); RP: receiving phase (acid aqueous solution).

Liquid membranes are systems made up of three immiscible phases: an aqueous source phase, which contains the chemical species of interest for valorization or removal from the system, an organic membrane phase that ensures the selective transport of the considered chemical species and an aqueous receiving phase in which it is immobilized [1,2,3]. Liquid membranes are usually differentiated based on the amount and form in which the membrane phase is found in the system and comprise volume liquid membranes (bulk liquid membranes, BLMs), liquid membranes on support (supported liquid membranes, SLMs) and emulsion liquid membranes (ELMs) [4,5,6]. Liquid membranes have been continuously developed because they ensure transport selectivity, allow for chemical reactions in the source, membrane and receiving phases, and can be made following various designs in order to meet process requirements (small investments, low productivity, large contact surfaces, easy operation, etc.) [7,8,9,10]. From the scale-up point of view, SLMs and ELMs (Figure 1a,b) are in direct competition in order to optimize the contact surface between the phases, the stability of the membrane and the losses of membrane material in the aqueous phases, and the recovery of the solvent membrane and of the chemical species of interest [11,12,13]. Liquid membranes, usually those based on organic solvents, consist of a pure solvent, a solution or a dispersion in which the continuous phase is the membrane solvent [14,15]. The chemical species that are added to the membrane solvent mainly have the role of transporters [16], but, more and more often, they also ensure the catalysis of a reaction process that takes place in the membrane to favor the separation in the desired chemical form of the target chemical species (Figure 1c) [17].

In this work, the target chemical species is p–nitrophenol, both because it can be easily reduced with molecular hydrogen and because this reaction can be observed through accessible means (UV–Vis, from yellow to colorless) [18].

The reactive membrane systems recently tested for the reduction and separation of p–nitrophenol (pNP) from source aqueous solutions use polymers as membrane phases (Figure 1c) or medium-chain n–alcohols, in which nanoparticles are dispersed [19,20,21,22,23].

The source of metallic osmium is represented by the remains (waste) of osmium tetroxide (OsO_4_) recovered in a polar or non-polar solvent, which makes it usable as a homogenous medium in reactions that aim to obtain osmium nanoparticles through reductions [19,20].

The osmium recovery process involves fixing or removing oxygen from osmium tetroxide (Equation (1)) as follows:

OsO_4_ + Red → Os + Ox (1)

Of course, it is preferable that the reductant or its reaction products are not necessarily removed from the reaction mass [22,23,24,25,26].

Osmium nanoparticles obtained in this way have been used both in oxidation and reduction processes with membrane systems [19,20], but these proposed membrane systems did not meet the researchers’ expectations in terms of their impact. The reluctance that researchers have regarding the use of osmium as a catalyst refers in particular to its toxicity, which is related to the increased volatility of osmium tetroxide, as well as to the aggressiveness of this oxide in the human body, since tetroxide seems to react with the side chains of proteins [27,28,29]. Thus, because of concerns regarding the toxicity of osmium, but mainly due to its apparent reaction rate constant that is close to but lower than that of most nano-metric catalysts based on platinum metals, it has been used less often [19,20,30,31].

The emulsion membrane type system proposed in this paper (Figure 1b,c) is based on the reaction system from Figure 1d. Our choice of a system with an emulsion membrane is clearly different from the recently studied reaction systems [19,20,30,31]. Emulsion membranes ensure a large contact surface, a micrometric membrane film for mass transfer and chemical reactions [32,33,34,35,36,37,38], and the possibility of choosing some green solvents as membranes (saturated n–alcohols).

We chose to study osmium nanodispersion in n–dodecanol as the membrane phase compared to that of n–decanol [30] due to the lower volatility of n–dodecanol, which contributes to the reduction in membrane-phase losses in aqueous phases and the atmosphere. At the same time, more precautions related to maintaining the working temperature must be taken compared to n–decanol. The operating time in the emulsion membrane system is much lower than in bulk liquid membrane and supported liquid membrane systems.

On the other hand, osmium nanoparticles have been synthesized according to various procedures, depending on their destination [39,40,41,42].

In this paper, we study nanodispersions with osmium nanoparticles obtained by a synthesis from osmium tetroxide reduced with undecenoic acid in n–dodecanol (nDD) or n–decanol (nD) media. The catalytic activity was determined by the reduction of p–nitrophenol (pNP) to p–aminophenol (pAP) in an emulsion membrane system. In this case, the membrane in the emulsion membrane system is the osmium nanodispersion in the chosen organic solvent, the source phase is an aqueous solution of p–nitrophenol and sodium tetraborate and the receiving phase is an acidic aqueous solution. The operation of the proposed system takes place using both co-current and counter-current flow, with the pH gradient being a working variable.

## 2. Results

The obtained results in this work are systematized as follows:

- The morphological characterization of nanodispersions in n–dodecanol by transmission electron microscopy (TEM), scanning electron microscopy (SEM) and dynamic light scattering (DLS);
- The compositional characterization of nanodispersions in n–dodecanol performed by an energy-dispersive spectroscopy analysis (EDAX) and thermal analysis coupled with gas chromatography (GC) and Fourier transform infrared spectroscopy (TA–GC–FTIR);
- The determination of the process performances of the nanodispersions of osmium particles in n–decanol or n–dodecanol for the reduction of p–nitrophenol.

In this paper, the characterization data for n–decanol have not been emphasized except when strictly necessary, because they were recently reported by the co-authors [30].

### 2.1. Morphological Characterization of the Obtained Nanodispersions

**Figure 2:**
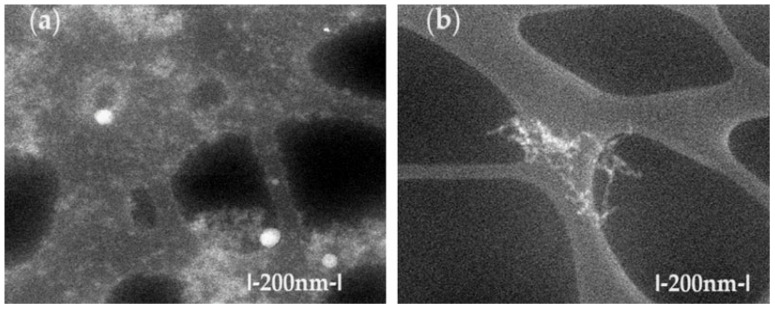
The scanning electronic microscopy (SEM) images for Os/n–dodecanol nanodispersions (**a**) before their use and (**b**) after their use in the reduction process of p–nitrophenol.

The TEM characterization of osmium nanodispersions in n–dodecanol was performed by washing them with ethanol, which we assumed would allow us to visualize the individual osmium nanoparticles. Unexpectedly, in the obtained images (Figure 2), the sample has an alveolar appearance with walls containing the osmium nanoparticles. We can state that, despite the sample’s preparation (washing the nanoparticles with ethanol), the osmium nanoparticles were still covered by the organic solvent. Thus, the observed aggregates separated in the alveolar walls containing n–dodecanol both before the p–nitrophenol reduction process (Figure 2a) and after processing (Figure 2b). Unfortunately, this organic coating prevented us from being able to increase the resolution of the images, because the sample shows the internal combustion of the alcohol with the osmium nanoparticles, which is practically highlighted by the disappearance of matter in the examined areas. The affected areas are presented as bright spots, confirming an observation previously described in the literature [30].

Although they were difficult to obtain, the images allowed for the observation of the aggregates of osmium nanoparticles as well as individual particles of about 10–50 nm.

**Figure 3:**
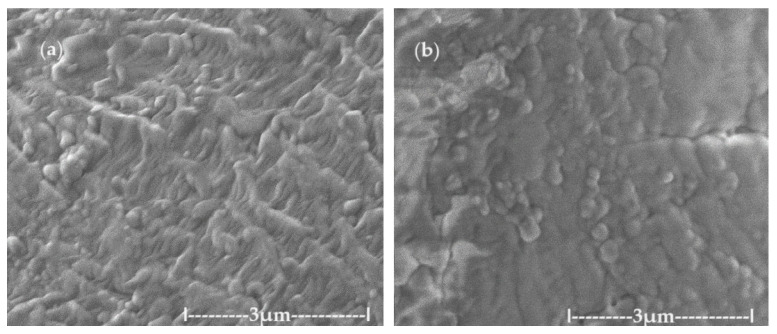
The images obtained by scanning electronic microscopy (SEM) for the primary Os/n–decanol dispersion (**a**) and for the Os/n–dodecanol dispersion (**b**) obtained after the p–nitrophenol reduction process.

In order to avoid the oxidation observed in the transmission electron microscopy (TEM) analysis, a scanning electronic microscopy (SEM) examination was attempted. Thus, the dispersion deposited on an aluminum support was dried in a vacuum and covered with a 50 nm gold film. Through this process, the overheating of the sample was avoided, and we obtained the morphology of the aggregates from the primary nanodispersion of osmium/n–dodecanol (Figure 3a) and those obtained after the p–nitrophenol reduction process (Figure 3b). After processing them in the p–nitrophenol reduction process, the morphology of the dispersions does not change drastically (Figure 3b), but the aggregates of the osmium nanoparticles are more easily visible.

In order to obtain the size of the osmium nanoparticles and their aggregates, a dynamic light scattering (DLS) analysis was performed after dispersing the sample in isopropyl alcohol.

**Figure 4:**
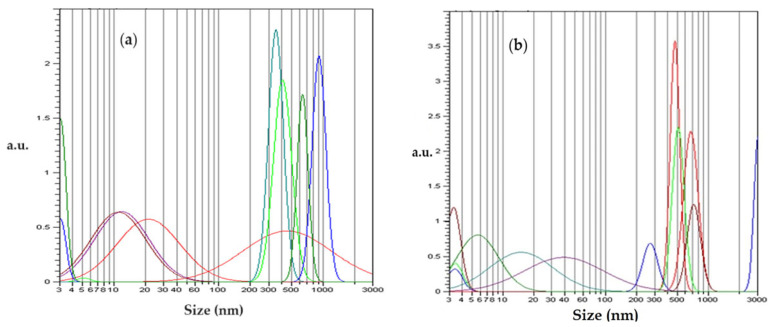
Size distribution of osmium nanoparticles in dispersion in isopropanol (**a**) before their use and (**b**) after their use in the p–nitrophenol reduction process.

The dynamic light scattering (DLS) analysis shows two-peak curves for some Gaussian-type distributions (Figure 4). The analysis of the dimensional distribution of the nanodispersion before its use in the reduction process of p–nitrophenol (Figure 4a) does not essentially differ from that of the dimensional distribution of the nanodispersion after the reduction process (Figure 4b). For the nanodispersion in isopropanol, two Gaussian distributions between 4 nm and 70 nm and between 200 nm and 1200 nm can be observed initially (Figure 4a). In the case of the second dispersion in isopropanol (Figure 4b), the Gaussian distribution widens at small sizes (from 4 nm to 170 nm) but narrows at large sizes (300 nm to 1000 nm).

The dimensional analyses of the nanodispersions of osmium nanoparticles in n–dodecanol provide the following information:

- Our transmission electron microscopy (TEM) analysis reveals agglomerations of nanoparticles from 10 nm to 30 nm both before and after the processing of the nanodispersions in the reduction process of p–nitrophenol;
- Our scanning electron microscopy (SEM) image analysis confirms the nanoparticle sizes in the nanodispersion;
- Our dynamic light scattering (DLS) analysis most relevantly indicates the size of nanoparticles in the range of 5 nm to 20 nm, as well as aggregates of nanoparticles with dimensions of 0.3 µm to 1.1 µm.

### 2.2. Compositional Characterization of the Obtained Nanodispersions

The compositional characterization of the dispersion of osmium nanoparticles had the following objectives:

- The determination of the composition and distribution of nanoparticles in the nanodispersion by an energy-dispersive spectroscopy analysis (EDAX);
- The determination of the composition of the solvents that remain in the nanodispersion after repeated washing with water by a thermal analysis coupled with gas chromatography and Fourier transform infrared spectroscopy (TA–GC–FTIR).

**Figure 5:**
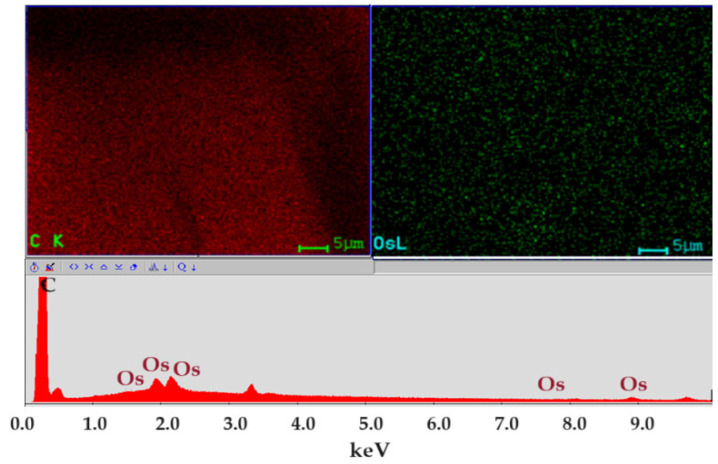
Energy-dispersive spectroscopy analysis (EDAX) for osmium nanodispersion in n–dodecanol.

Figure 5 shows the spectrum of the nanodispersion in which the carbon atoms generated by the organic solvents and elemental osmium are present. The distribution map of the two elements is uniform in the nanodispersion, which ensures its stability.

Thermal analyses (TG and DSC) were performed to determine the composition of the solvents in the nanodispersion. The analyses were carried out in an N_2_ atmosphere, so no oxidation of the organic substance or osmium was foreseen.

**Figure 6:**
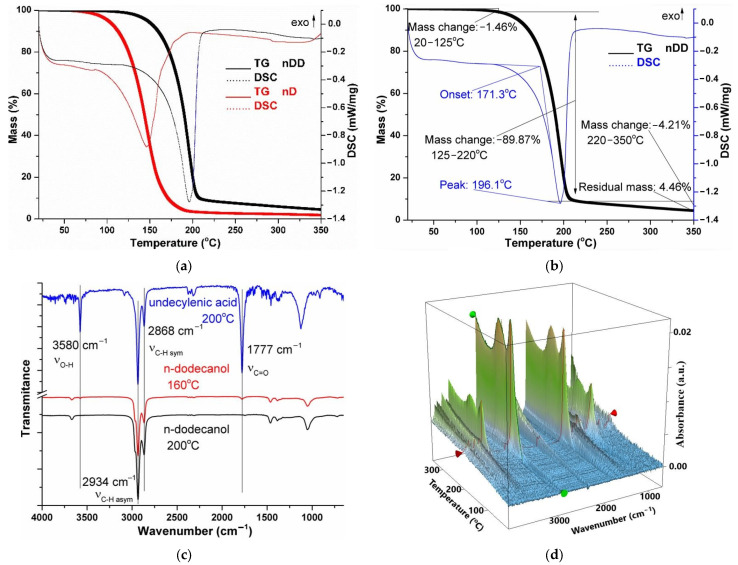
Thermal analysis (**a**,**b**) and FTIR analysis of decomposition gases: two-dimensional FTIR (**c**) and three-dimensional FTIR (**d**).

The Os/n–dodecanol (nDD) dispersion sample in comparison with the n–decanol (nD) dispersion sample (Figure 6a, Table 1) started to lose its liquid part over 125 °C, with evaporation between 125 and 220 °C representing a loss of 89.87% of its initial mass. The process is accompanied on the DSC curve by an endothermic effect with the minimum at 196.1 °C generated by the evaporation of the solvent, undecylenic acid, but with a boiling point that is lower than the value reported in the literature, i.e., 275 °C. The residual mass is 4.46% and consists of osmium compounds (Figure 6b).

In the two-dimensional and three-dimensional FTIR spectra for Os–nDD up to 200 °C (Figure 6c,d), the presence of CO_2_ is mostly seen at 2355 cm^−1^, traces of CO can be seen at 2169 cm^−1^, and water and the corresponding vibration of Csp^3^–H can be seen at 2964 cm^−1^. A small peak is observed at 3072 cm^−1^ corresponding to Csp^2^–H fragments (Figure 6c,d) [31].

As the temperature increases, the peak at 1721 cm^−1^ is attributed to the C=O bond from the undecylenic acid, which is eliminated at higher temperatures (Figure 6c) but is not completely consumed when preparing the dispersion via the reduction [20,30,31].

The compositional characterization of the osmium nanoparticle nanodispersions in n–alcohols shows the following:

- Osmium nanodispersions in n–dodecanol have an elemental composition (EDAX) that indicates the presence of carbon and osmium;
- The distribution map (EDAX) of the two elements, osmium and carbon, shows uniformity on the surface;
- Our thermal analysis coupled with gas chromatography and Fourier transform infrared (TA–GC–FTIR) spectroscopy for the n–dodecanol-based nanodispersion highlights the presence of n–decanol, as well as unreacted undecylenic acid.

### 2.3. Determining the Process Performances for p–Nitrophenol Reduction

The chosen chemical species for conducting the experiments is p–nitrophenol, for which the specialized literature provides numerous conclusive data [19,20,30,31]. At the same time, p–nitrophenol is a toxic substance that is easy to follow spectrophotometrically via its reductions in p–aminophenol [20,30,31,32,33,34,35,36,37,38,39]. In this paper, the accuracy of our determinations is ±0.3%, imposed both by our method of sampling and preparation of the samples, as well as by the interaction of the chemical species dissolved in the aqueous phase with nitrophenol (alkaline medium, sodium ions, n–alcohols).

The experiments show the results of p–nitrophenol’s reduction to p–aminophenol using an ELM, which contains the acidic aqueous receiving phase (blue) found inside the nanodispersion emulsion containing osmium nanoparticles (yellow) and an aqueous basic source phase (green) containing p–nitrophenol and sodium borohydride outside of the emulsion (Figure 1).

In our case, the temperature at which the studies were carried out was 24 ± 1 °C.

Although, according to the Arrhenius equation, our studies should have included an operating temperature variation, this was not done for two reasons:

- Increasing the temperature favors the volatility of alcohols and therefore results in losses of the membrane solvent;
- The stability of the emulsion membrane decreases with the increasing temperature.

Temperatures lower than that in the laboratory were not taken into account due to technical complications and the increase in the viscosity of alcohols, thus the decrease in mass transfer.

**Figure 7:**
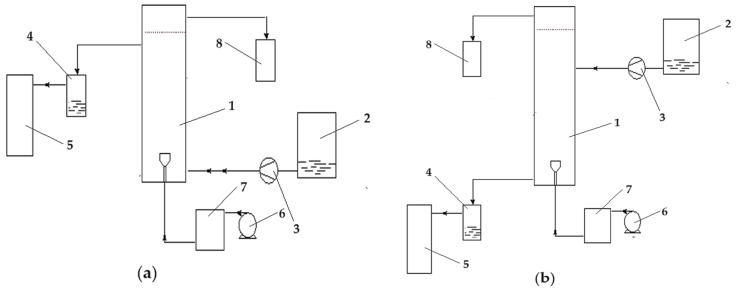
Phase circulation in the column for the reduction of p–nitrophenol to p–aminophenol: (**a**) co-current and (**b**) counter-current. 1—the reaction column; 2—source-phase (SP) tank; 3—source-phase metering pump; 4—source-phase level maintenance vessel; 5—source-phase tank; 6—emulsion dosing pump; 7—intermediate emulsion vessel; and 8—emulsion collector vessel.

The reduction reaction takes place in a column-type reactor fed at the base with the emulsion containing the receiving phase, and the source phase is introduced either at the base of the column (co-current) (Figure 7a) or at the top (counter-current) (Figure 7b).

The conversion (η%) or extraction efficiency (*EE*%) for the species of interest using the concentration of the solutions [19,20,21] was calculated as follows:

(2) $$\eta \%\;or\;EE\left(\%\right)=\frac{\left(c_{0}-c_{f}\right)}{c_{0}}\cdot 100$$

where *c_f_* is the final concentration of the solute (the considered chemical species) and *c*_0_ is the initial concentration of the solute (the considered chemical species).

The same extraction efficiency can also be computed based upon the absorbance of the solutions [30,31] as follows:

(3) $$\eta \%\;or\;EE\left(\%\right)=\frac{\left(A_{0}-A_{s}\right)}{A_{0}}\cdot 100$$

where *A*_0_ is the initial absorbance of the sample solution and *A_s_* is the current absorbance of the sample.

At a pH higher than 10 of the source phase and a pH less than 4 of the receiving phase, the results for the conversion (*η*) of p–nitrophenol to p–aminophenol, with the emulsion based on an alcoholic nanodispersion containing osmium nanoparticles, are superior to those presented previously [20,30,31] using supported liquid membranes (SLMs) or bulk liquid membranes (BLMs). This observation led us to study the conversion of p–nitrophenol with emulsion membranes based on osmium nanoparticles and n–decanol or n–dodecanol.

**Figure 8:**
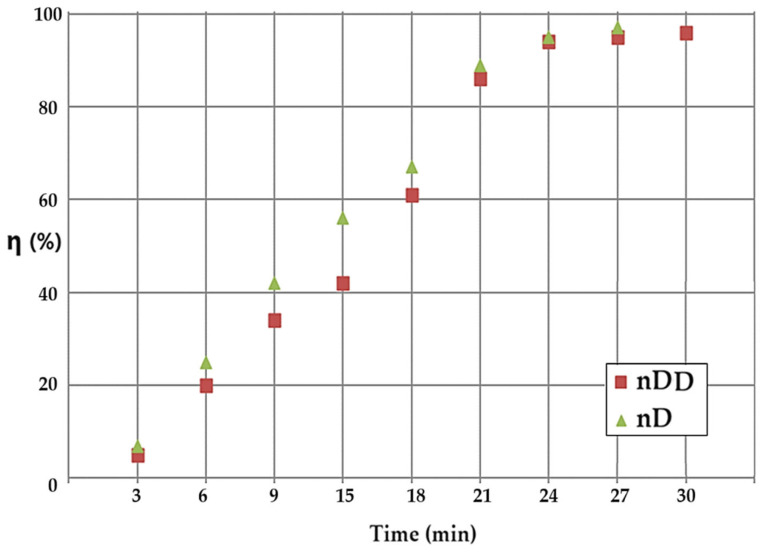
Comparison of the results, as a function of time, obtained from the conversion of p–nitrophenol with the nanodispersion of n–dodecanol (brown squares) and those for n–decanol (green triangles) in counter-current operating system, respectively.

The current data are presented by comparing the results obtained with the nanodispersion of n–dodecanol (the curve with the brown squares) and those for n–decanol (the curve with the green triangles) (Figure 8) in a co-current system, respectively. Throughout the process, the conversion obtained with the nanodispersion based on n–decanol is superior to that based on n–dodecanol in the average operating range of 6–18 min. The flattening of the conversion value after 20 min of operation is related to the exhaustion of the working reagents.

**Figure 9:**
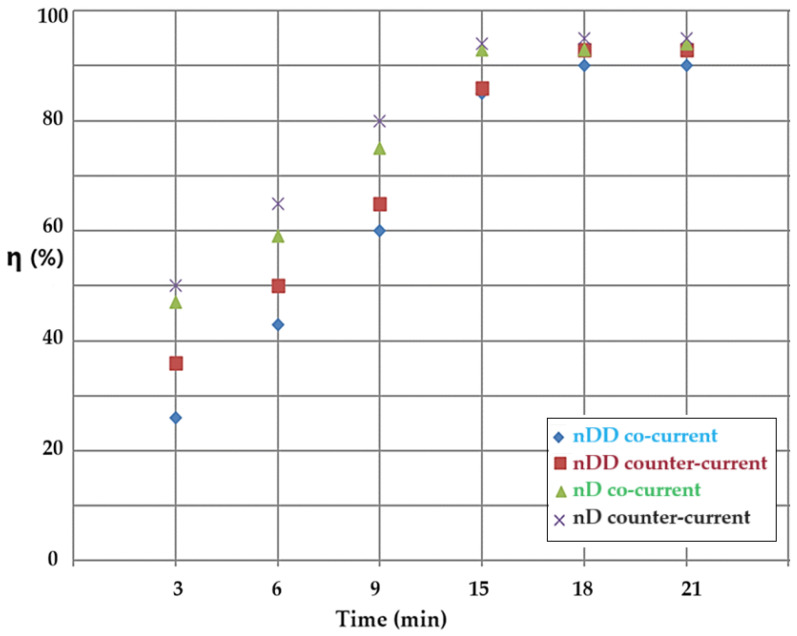
Comparison of the results obtained during the conversion (η) of p–nitrophenol with those of the nanodispersion of n–dodecanol (blue diamond, brown squares) and of n–decanol (green triangle and purple crosses), respectively, depending on time and the flow mode of the phases in the stripping column: counter-current (brown square and purple crosses) and co-current (blue diamonds and green triangles) systems.

In the stripping column with emulsion membranes containing drops of the aqueous phase at a pH of 2 and the source aqueous solution at a pH of 12, the conversion (η) was studied depending on the type of the membrane solvent and on the flow mode of the phases. Over the entire the working range, the conversion results obtained with membranes based on n–decanol (the green triangles and black crosses) are superior to those obtained with the dispersion in n–dodecanol (the blue diamonds and brown squares) (Figure 9). At the same time, the results of the conversion obtained in the counter-current flow system (the brown squares and black crosses) are superior to those obtained by the recirculation of the phase in the co-current system (the blue diamonds and green triangles) (Figure 9).

The operation of the counter-current reaction system ensures a relatively equal concentration gradient of the reactants during the reaction and mass transfer, while in the case of the co-current system, the concentration gradient decreases constantly [42]. After approx. 15 min of operation, when the concentration difference of the reactants is minimal, the capping effect of the conversion appears. Basically, when a high conversion rate is desired, the counter-current operation is preferable when the working time is minimized. If the operating time is not an impediment to the reaction and mass transfer process, then either type of flow can be chosen.

**Figure 10:**
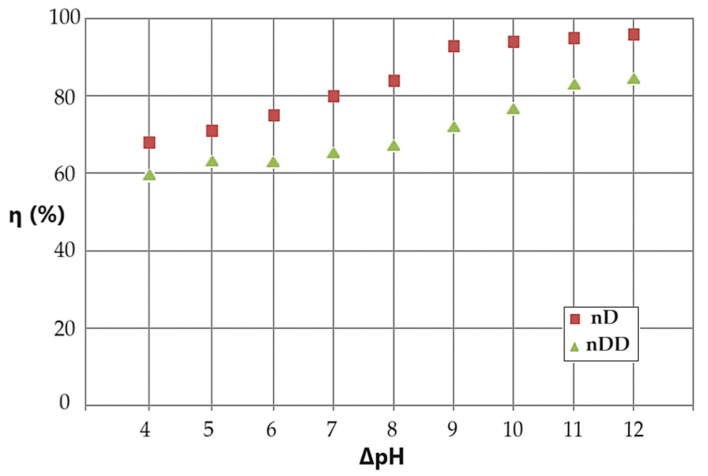
Results of the conversion (η) depending on the pH difference between the source and receiving phases, which circulate in counter-current system for the emulsion based on n–decanol (brown squares) or n–dodecanol (green triangles).

By choosing the experimental variant with the circulation of the aqueous phase, the basic source in the counter-current system, with the nanodispersion based on n–decanol or n–dodecanol containing drops of the acidic aqueous phase, the conversion variation (η) could be obtained according to the pH difference between the aqueous phases at an operating time constant of 15 min (Figure 10). The pH difference between the source and receiving phases is achieved with aqueous solutions of sodium hydroxide and sodium tetrahydride, respectively, with hydrochloric acid and ultrapure water (Table 2). The pH difference is measured before processing. During the reduction of p–nitrophenol and the transport of p–aminophenol, the technical conditions, in particular the distribution of n–alcohols on the surface of the measuring electrode, cannot be monitored, including the pH value and its gradient.

The conversion of p–nitrophenol to p–aminophenol increases with the increase in the pH difference between the aqueous phases of the membrane system (Figure 10). Thus, if the pH difference between the source and receiving aqueous phases is 4 units, then we have a conversion of 69%, and at a difference of 12 units, we have a conversion of 98% for n–decanol and from 60% to 85% for n–dodecanol, respectively. Practically, if we aim for an optimal conversion, then the difference in pH values between the aqueous phases must be at least 9 units.

**Figure 11:**
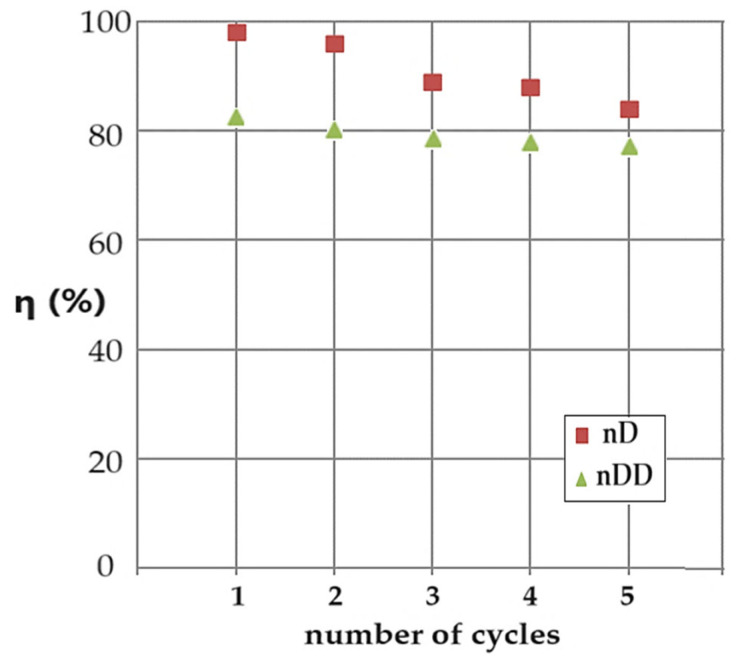
Conversion results (η) depending on the number of conversion cycles with the same emulsion based on osmium nanoparticles in n–decanol (brown squares) or n–dodecanol (green triangles), containing the receiver phase at a pH of 2 and the source phase at a pH of 12 containing p–nitrophenol and refreshed sodium borohydride, which circulates in the counter-current system.

An important aspect of the study of catalytic materials is the regeneration problem. Particularly in cases of membrane-type heterogeneous catalysis, this aspect must be treated carefully. Figure 11 shows the conversion (η) values for five reuse cycles of the ELM based on osmium nanodispersions in n–decanol. The cyclic catalysis process was carried out with the emulsion based on the osmium nanoparticles in n–decanol in the counter-current system with the source phase at a pH of 12. The receiving aqueous phase at a pH of 2 is recirculated as such by means of the nanodispersion. The source phase at a pH of 12 is refreshed for each reaction cycle with an equal concentration of p–nitrophenol and sodium borohydride.

The decrease in conversion by almost twenty percent, depending on the number of contact cycles of the phases, is caused by the increase in pH in the receiving phase, which leads to a decrease in the pH differences between the phases, although the pH of the source phase remains relatively constant at 12. In the case of dodecanol (Figure 11), the initial conversion is lower (approx. 83%), but the decrease depending on the number of cycles is smaller, at approx. 78%. Practically, if the catalytic emulsion is to be reused, the one based on n–dodecanol is more advantageous, and if a higher conversion rate is desired, the one based on n–decanol is used, but the number of reuse cycles will be reduced.

The catalytic activity of both systems decreases because the osmium nanoparticles aggregate, thus lowering, for each operating cycle, the interphase contact surface. After five cycles of use, the decrease in catalytic activity requires the regeneration of the membrane nanodispersion.

**Figure 12:**
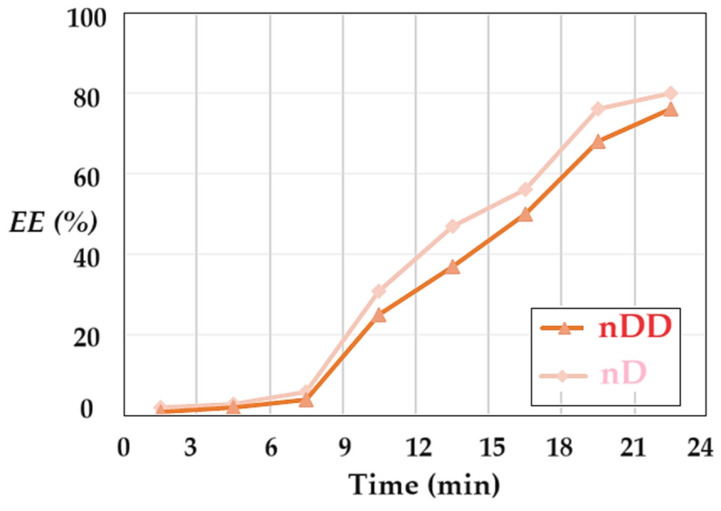
The separation efficiency (EE) of p–aminophenol from the emulsion formed by osmium nanodispersion in n–dodecanol or n–decanol and the receiving aqueous phase at a pH of 2, depending on the operating time.

The extraction efficiency (*EE*) of p–aminophenol was determined by separating the emulsion membrane in the nanodispersion containing osmium nanoparticles in n–dodecanol and the receiving aqueous phase at a pH of 12. For the separation of the phases from the emulsion (the collapse or breaking of the emulsion), a membrane based on cellulose acetate was used in a Sartorius ultrafiltration module of a dead-end filtration unit. The results for p–aminophenol’s extraction efficiency are presented in Figure 12. It is noted that the separation efficiency starts with low values in the first time interval, then it develops rapidly until reaching values of 75–80%. The separation efficiency with the n–decanol membranes is superior over the entire time interval to that with the n–dodecanol-based membranes.

The delay in the separation of p–aminophenol compared to the conversion of p–nitrophenol to p–aminophenol can be justified both by the diffusion process through the liquid membrane and by the slow transfer from the liquid membrane to the acidic environment of the receptor phase. The extraction efficiency value is lower than the conversion value at the same operating time.

The process of reducing p–nitrophenol to p–aminophenol with osmium nanodispersions in n–decanol and n–dodecanol in the liquid membrane emulsion system shows the following:

The liquid membrane emulsion system consists of the following:

- The aqueous-source phase with an alkaline pH, containing p–nitrophenol and sodium borohydride;
  1. The membrane phase—the dispersion of osmium nanoparticles in n–decanol or n–dodecanol;
  2. The receiving phase solution with an acidic pH.
- The installation (working plant) allows for the co-current or counter-current circulation of the phases, the basic source and the emulsion, which contains drops of the acidic aqueous solution in the osmium nanodispersion in n–decanol or n–dodecanol;
- The system that operates with the nanodispersion in n–decanol ensures a conversion rate of p–nitrophenol to p–aminophenol that is higher than that of the nanodispersion in n–dodecanol;
- The counter-current operation of the phases leads to higher conversion rates than the co-current operation;
- At the same operating time, the increase in the pH difference between the source and receiving aqueous phases leads to the increase in the conversion of p–nitrophenol to p–aminophenol;
- Reusing the catalytic emulsion containing the osmium nanodispersions in n–alcohols in the counter-current flow regime decreases the conversion value from approx. 98% in the first cycle to approx. 83% in the fifth cycle for n–decanol and from 60% to 85% for n–dodecanol;
- The p–aminophenol separation efficiency is below the p–nitrophenol conversion value over the entire operating time interval.

## 3. Discussion

Various catalytic materials for the reduction of p–nitrophenol to p–aminophenol have been recently reported and have very good results both in terms of conversion and catalyst regeneration [43,44,45,46,47,48,49,50,51].

In this work, catalytic systems with results comparable to the results obtained in the Os–NP/n–decanol or n–dodecanol emulsion membrane system were selected ([52,53,54,55,56,57,58,59], Table 2).

The data in Table 3 were calculated similarly to the comparison data, taking into account the most probable kinetic equation (Equation (4)) [30,31,52,53,54,55,56,57,58,59] as follows:

(4) ln (CC0)=−k·K·t=−kapp·t

where *C* and *C*_0_ are the concentrations (in mg/L) of pNP at *t =* 0 and *t* ≠ 0, respectively, with *t* being the reaction time, *k* being the reaction rate constant (mg/(L×min)), *K* being the adsorption coefficient of the reactant (L/mg) and *k_app_* (s^−1^) being the apparent catalytic rate constant when the concentration (*C*_0_) is very low [60,61].

The reaction kinetics show that the pNP’s reduction is a pseudo-first-order reaction [30,31,60,61].

Catalytic systems based on osmium nanoparticles, coupled with polymer or liquid membrane processes, have been less frequently reported [19,20,21,30,31]. In these hybrid membrane–catalytic processes, p–nitrophenol is reduced to p–aminophenol with osmium nanoparticles. This hybrid process involves both the conversion of p–nitrophenol in the source phase and the separation of p–aminophenol in the receiving phase through a membrane process. Previously, bulk liquid membranes based on osmium nanoparticles were tested [20,21,30], as well as liquid membranes on support (SLMs) [19,31].

The emulsion membrane based on the nanodispersion of osmium in n–decanol or n–dodecanol has a superior performance both in the conversion of p–nitrophenol and in the separation of p–aminophenol from the system. Taking into account relation (4), an apparent constant (*k_app_*) was obtained, with maximum values between 0.1 × 10^−3^ s^−1^ for dodecanol and 0.9 × 10^−3^ s^−1^ for decanol. These values are about an order of magnitude higher than the other systems with membranes based on osmium nanoparticles that have been previously reported [19,20,30,31], but below the values reported in the specialized literature for catalysts based on nanoparticles obtained from the platinum group [52,53,54,55,56,57,58,59].

**Figure 13:**
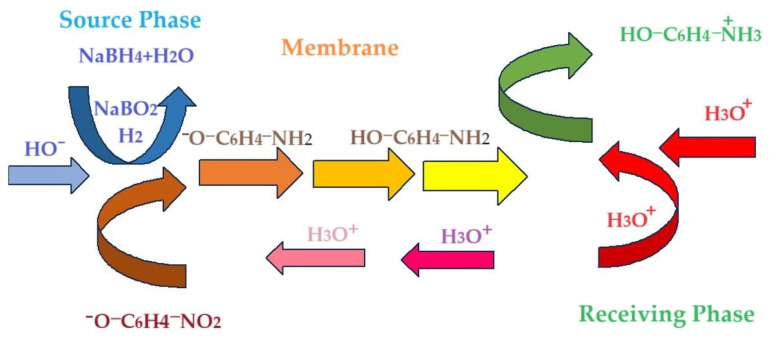
Schematic representation of the hybrid process of p–nitrophenol catalytic reduction and p–aminophenol membrane recovery.

Figure 13 shows schematically the hybrid process of the catalytic reduction of p–nitrophenol and the membrane recovery of p–aminophenol through the new emulsion-type membrane.

For the conversion of p–nitrophenol and the efficiency of p–aminophenol separation according to the scheme in Figure 13, the following can be taken into consideration: the different molar masses of n–alcohols, mutual solubility of n–alcohols in water and of water in n–alcohols, viscosity, relative polarity and pKa values (Table 4).

Through the proposed mechanism, two interfaces (as follows):

1. The source phase/organic phase interface, containing osmium nanoparticles;
2. The organic phase/aqueous receiving phase interface.

and three phases (as follows):

1. Aqueous source phase;
2. Organic phase containing osmium nanoparticles;
3. Receiving aqueous phase.

are considered responsible for the conversion of p–nitrophenol and the transport of p–aminophenol in water.

A simplified model of the conversion of p–nitrophenol and the transport of p–aminophenol involves five steps as follows:

- The diffusion of p–nitrophenolate and molecular hydrogen from the source aqueous phase to the interface with the catalytic organic phase due to the content of osmium nanoparticles.
- The penetration of the source aqueous phase/organic phase interface simultaneously with the conversion of p–nitrophenolate to p–aminophenol;
- The diffusion of p–aminophenol across the membrane to the organic phase/receiving aqueous phase interface;
- The penetration of the organic phase/receiving aqueous phase interface simultaneously with the reaction of p–aminophenol with the proton;
- The diffusion of protonated aminophenol in the receiving aqueous phase.

In the proposed model, all the parameters of the working system favor the n–decanol-based membrane (Table 3), as follows:

- The mutual solubility of water in n–alcohols is higher for n–decanol compared to that for n–dodecanol by almost an order of magnitude;
- The viscosity of n–decanol is lower by about 30% compared to that of n–dodecanol.

The two presented aspects lead to friendlier interfaces between the organic phase (n–alcohol) and the two aqueous phases (source and receiver), respectively. This means that the interfaces with n–decanol contain more water in n–decanol and more n–decanol in water, favoring the diffusion and penetration of the interface by p–nitrophenol and the source of molecular hydrogen. Of course, with the parameters of n–decanol (solubility and viscosity) being an order of magnitude higher than those of n–dodecanol, the conversion of p–nitrophenol and the transport of p–aminophenol are not favored.

The proposed model responds to the results presented previously (Figure 8, Figure 9 and Figure 12), which are better for the membrane containing n–decanol than the one based on n–dodecanol.

From a practical point of view, the only deficiency of n–decanol is that it remains in the aqueous phases at a maximum concentration of 0.037 g/L compared to n–dodecanol at a concentration of 0.004 g/L. In terms of technological exploitation, this observation leads to the design of a method for the elimination of n–decanol from aqueous effluents. The two n–alcohols with an even number of carbon atoms are biodegradable, which would solve this problem via bio-degradation.

Compared to heterogeneous catalytic systems with solid catalysts, systems with the dispersion of osmium nanoparticles in n–alcohols differ in the type of interface used: adsorption in the first case, and absorption in the second. Depending on the nature of the nitroderivative, either heterogeneous adsorption catalysis or heterogeneous absorption catalysis can be favored.

A favorable aspect of the chosen working method is the reception of p–aminophenol resulting from the reduction of p–nitrophenol in the receiving phase at a concentration that is 10 times higher. This concentration factor would create the possibility of using the p–aminophenol solution as such in the pharmaceutical or dye industries.

If, in terms of reductions, osmium-based membrane systems are inferior to other catalyst systems, it would be interesting for these systems to be used in selective oxidation processes.

## 4. Materials and Methods

### 4.1. Reagents and Materials

All reagents used in the presented work were of analytical grade. The following were purchased from Merck (Merck KGaA, Darmstadt, Germany): osmium tetroxide, sodium hydroxide, hydrochloric acid, NaBH_4_, p–aminophenol and p–nitrophenol.

The following membrane components were purchased from Sigma-Aldrich (Merck KGaA, Darmstadt, Germany): t–butyl alcohol, n–octanol, n–dodecanol and 10–undecylenic acid (undecenoic acid), with the characteristics presented in Table S1.

Purified water characterized by a conductivity value of 18.2 μS/cm was obtained with an RO Millipore system (MilliQ^®^ Direct 8 RO water purification system, Merck, Darmstadt, Germany).

### 4.2. Methods and Procedures

#### 4.2.1. Analytical Methods

For the dynamic light scattering (DLS) analysis [30], we used the following granulometer equipment: Coulter N4 Plus (laser He–Ne, 632.8 nm) (Beckman Coulter GmbH, Krefeld, Deutschland).

For transmission electron microscopy (TEM), we used a high-resolution 80–200 kV Titan THEMIS transmission microscope (Thermo Fisher Scientific, former FEI, Hillsboro, OR, USA) equipped with an Image Corrector and EDXS detector in the column. The microscope was operated at 200 kV in transmission mode [30,31].

The scanning electron microscopy studies, SEM and HFSEM, were performed on a Hitachi S4500 system (Hitachi High-Technologies Europe GmbH, Krefeld, Germany) [21,22].

Thermal analyses (TG–DSC) were performed with an STA 449C F3 apparatus from Netzsch (Selb, Germany) with an FTIR Tensor 27 from Bruker (Bruker Co., Ettlingen, Germany) [19,20,21].

The UV–Vis studies were performed using the following dual-beam UV equipment: Varian Cary 50 (Agilent Technologies Inc., Santa Clara, CA, USA) at a resolution of 1 nm, spectral bandwidth of 1.5 nm and a scan rate of 300 nm/s. [19].

The UV–Vis validation analysis of the p–nitrophenol solutions was performed on a CamSpec M550 spectrometer (Spectronic CamSpec Ltd., Leeds, UK) [21,23].

The electrochemical analysis was followed up with a PARSTAT 2273 Potentiostat (Princeton Applied Research, AMETEK Inc., Oak Ridge, TN, USA) [19,20].

The pH, conductance and anion concentration (in the source phase or in the receiving phase) were determined using a conductance cell or combined selective electrode (HI 4107, Hanna Instruments Ltd., Leighton Buzzard, UK) and a multi-parameter system (HI 5522, Hanna Instruments Ltd., Leighton Buzzard, UK) [19,22].

#### 4.2.2. Preparation of Nanodispersion of Osmium Nanoparticles in n–Dodecanol and n–Dodecanol

The preparation of nanodispersion of osmium nanoparticles in n–decanol was previously presented [30]. Briefly, to obtain the dispersion of osmium nanoparticles, dissolve 1 g (0.0039 mol) of osmium tetroxide in 50 mL t–butanol at room temperature in a 100 mL conical vessel.

Separately, in a 2000 mL vessel with a hemispherical bottom, add 1250 mL (1000 g) of n–dodecanol or n–decanol to 7.249 g (0.039 mol) of 10–undecylenic acid. After approx. 10 min of homogenization, the osmium tetroxide solution is added in drops, and it is possible to observe the instant formation of an intensely black dispersion. The dispersion obtained (Os–NP/nDD or Os–NP/nD) is washed five times with 200 mL of pure water, which is analyzed spectrophotometrically to track the removal of t–butanol or other organic components.

The stability of the prepared nanodispersion is monitored by putting it in contact with 200 mL of pure water for two weeks, with the aqueous layer and the osmium dispersion at the water interface being analyzed spectrophotometrically with a Motic microscope daily (MoticEurope, S.L.U., Barcelona, Spain).

#### 4.2.3. Preparation of Emulsion of Acidic Aqueous Solution (Receiving Phase) in n–Alcohols

A stock emulsion is prepared by intensively mixing 500 mL of osmium dispersion in n–alcohol and 500 mL of acidic aqueous solution. The intense stirring of the immiscible phases is carried out with a propeller-type stirrer (Figure S1) with 150 rotations per minute. A black dispersion is obtained whose conductance is lower than that of ultrapure water.

#### 4.2.4. Reduction of p–Nitrophenol to p–Aminophenol

The source phase consisting of a 2.78 g/L (2 × 10^−2^ mol/L) solution of p–nitrophenol in ultrapure water is mixed with 7.566 g/L (0.2 mol/L) of a freshly prepared solution of sodium borohydride.

A volume of 1000 mL of this solution is introduced into the 1500 mL reaction column (Figure S2a) and recirculated by means of a peristaltic pump, with a flow rate of 15.0 mL/s. The source solution is recirculated either co-currently or counter-currently (Figure 11) with 200 mL of emulsion containing the receiving phase (100 mL) embedded in the osmium nanodispersion in n–alcohols (100 mL). The dispersion of the emulsion containing the receptor phase is carried out with a specific device (Figure S2b) with the help of the peristaltic pump with a flow rate of 3.5 mL/s.

The progress of the reaction is monitored by taking 1.0 mL of the source solution at predetermined operation interval and analyzing it spectrophotometrically at λ = 404 nm. The conversion or separation efficiency is calculated by relations (3) and (4).

## 5. Conclusions

Osmium nanoparticle reaction systems placed on membranes (polymeric or composite) or bulk membranes have recently been studied to highlight their catalytic characteristics in reduction reactions of p-nitrophenol to p-aminophenol.

This paper presents the results of a reaction system with osmium nanoparticles dispersed in n–decanol or n–dodecanol, which is constituted in an emulsion-type membrane.

The osmium nanoparticles in the membrane dispersion were characterized by SEM, TEM, EDAX and AT (TG and DSC), and a complex analysis of the gases during the thermal decomposition of the dispersion (TA–GC–FTIR) was also performed. The chemical species taken as the target substance is p–nitrophenol. The source phase of the membrane system consists of p-nitrophenol dissolved in an aqueous solution of sodium borohydride. After the reduction in the membrane phase, the p-aminophenol formed is immobilized as an ammonium ion in an acidic receptor solution.

In the proposed system, the flow regime (co-current or counter-current) of the membrane dispersed through the source phase, the pH difference between the source and receiving phases, the number of operating cycles and the nature of the n–alcohol membrane were varied.

The obtained results reveal that the counter-current operation is more advantageous than the co-current operation until a certain level is reached, which is given by the co-current flow. In the middle of the working time interval, the counter-current operation is preferred.

The number of reuse cycles of the emulsion membrane is limited by the decrease in conversion. Practically, after five cycles of use, the membrane dispersion must be regenerated. In all experimental cases, the conversion results are higher for n–decanol than for n–dodecanol.

Increasing the pH difference between the source and receiving aqueous phases leads to an increased conversion rate. Under the most favorable working conditions, the apparent constant (*k_app_* (s^−1^)) is 0.1 × 10^−3^ for dodecanol and 0.9 × 10^−3^ for decanol.

## Figures and Tables

**Table 1:** The thermal characteristics of the dispersion diagrams of osmium nanoparticles in n–decanol and n–dodecanol.

Sample	Mass Loss up to	Solvent Removal	Endo Peak	Residual Mass
n–decanol	1.96% at 95 °C	94.94% between 95–220 °C	156.8 °C	1.70%
n–dodecanol	1.46% at 125 °C	89.87% between 125–220 °C	196.1 °C	4.46%

**Table 2:** Achieving of pH difference between the source and receiving phases.

pH Receiving phase	6	5	5	4	4	3	3	2	2
pH Source phase	10	10	11	11	12	12	13	13	14
ΔpH	4	5	6	7	8	9	10	11	12

**Table 3:** Comparative data of the apparent catalytic rate constant (*k_app_*) in the catalytic reduction reaction of p–nitrophenol to p–aminophenol.

Catalytic Material	*k_app_* (s^−1^)	Year	Refs.
Os-nanoparticles on polypropylene hollow fiber membranes	2.04 × 10^−4^–8.05 × 10^−4^	2022	[30]
Osmium nanoparticles/n–decanol bulk membrane	0.8 × 10^−4^–4.9 × 10^−4^	2022	[31]
Plasma-enabled synthesis of Pd/GO rich in oxygen-containing groups and defects	13.9 × 10^−3^	2022	[52]
Immobilizing of palladium on melamine functionalized magnetic chitosan beads	16.5 × 10^−3^	2021	[53]
Ultra-small iridium nanoparticles as active catalysts	5.3 × 10^−3^	2020	[54]
Pd@MIL–100(Fe) composite nanoparticles as efficient catalyst	6.5 × 10^−3^	2018	[55]
Highly efficient Pd/UiO–66–NH_2_ film capillary microreactor	62.3 × 10^−3^	2017	[56]
Magnetic nano-porous PtNi/SiO_2_ nanofibers	12.84 × 10^−3^	2017	[57]
Iridium (0), platinum (0) and platinum (0)–iridium (0) alloy nanoparticles	0.41 × 10^−3^ (Pt) 0.21 × 10^−4^ (Ir)	2017	[58]
Iridium oxide nanoparticles and iridium/iridium oxide nanocomposites	2.5 × 10^−3^–5.5 × 10^−3^	2015	[59]
Emulsion membranes based on Os–NP/n–decanol or n–dodecanol	0.1 × 10^−3^–0.9 × 10^−3^	This work	

**Table 4:** The main properties of membrane n–alcohols.

Organic Compounds	Molar Mass (g/Mol)	Solubility in Water (g/L)	Water Solubility (g/L)	Viscosity (cP)	Relative Polarity Measure	pKa
n–decanol (nD)	158.28	0.037	0.0211	12.05	−0.540	15.21
n–dodecanol (nDD)	186.34	0.004	0.0019	18.80	−0.511	16.84

## Data Availability

Data are contained within the article.

## Supplementary Materials

The following supporting information can be downloaded at: https://www.mdpi.com/article/10.3390/molecules29081842/s1, Figure S1: Helix propeller stirrer for dispersing osmium nanoparticles in n–alcohols: (a) overview; (b) detail of the stirring component; Figure S2: The p–nitrophenol reduction reaction column (a); and the detail of the emulsion (b); Table S1: The characteristics of the substances used for the emulsion liquid membrane preparation.
