# Supplementary material for: Emulsion Liquid Membranes Based on Os–NP/n–Decanol or n–Dodecanol Nanodispersions for p–Nitrophenol Reduction
Source: Molecules. 2024 Apr 18;29(8):1842. doi: 10.3390/molecules29081842 (PMC11055161; doi:10.3390/molecules29081842)
Supplement: Supplementary file 1 [file molecules-29-01842-s001.zip › molecules-2924575-supplementary.pdf]

Supplementary Material

Table S1. The characteristics of the substances used for the emulsion liquid membrane preparation.

Component	Chemical formula	Molar mass (g/mol)	Density (g/cm³)	pKa	Solubility in water (g/L)	λ (nm)
Osmium tetroxide	OsO_4	254.23	4.91	-	soluble	-
Sodium borohydride	$Na BH_4$	37.83	1.07	alkaline aqueous solution	soluble	-
t–butyl alcohol	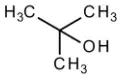	74.12	0.775	16.54	miscible	-
p–Nitrophenol (pNP)	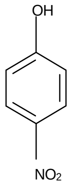	139.17	1.48	7.1	16.0	317 (phenol) 404 (phenolate)
p–Aminophenol (pAP)	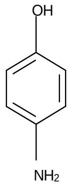	109.13	1.13	5.5<br>10.3	15.0	317
n–decanol (nD)	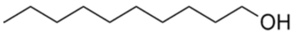	158.28	0.830	15.21	0.037	197
n–dodecanol (nDD)	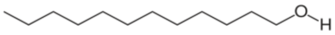	186.34	0.8831	16.84	0.004	201
10–undecylenic acid	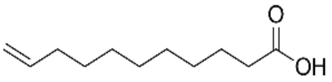	184.28	0.912	5.02	0.074	-

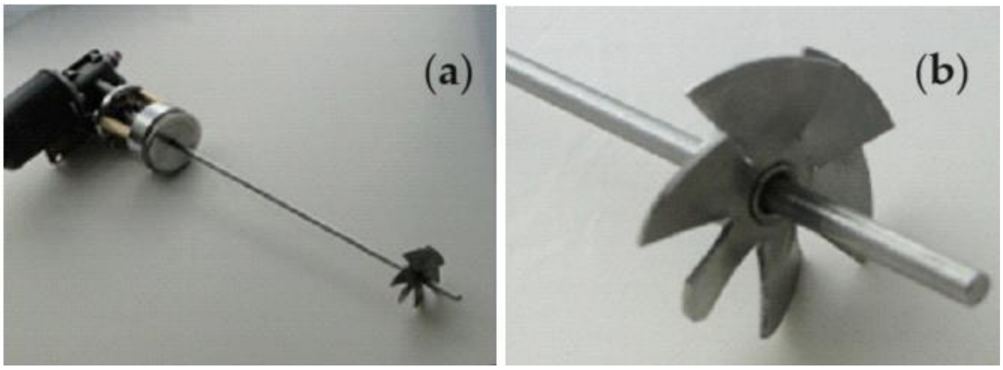

Figure S1. Helix propeller stirrer for dispersing osmium nanoparticles in n–alcohols: **(a)** overview; **(b)** detail of the stirring component.

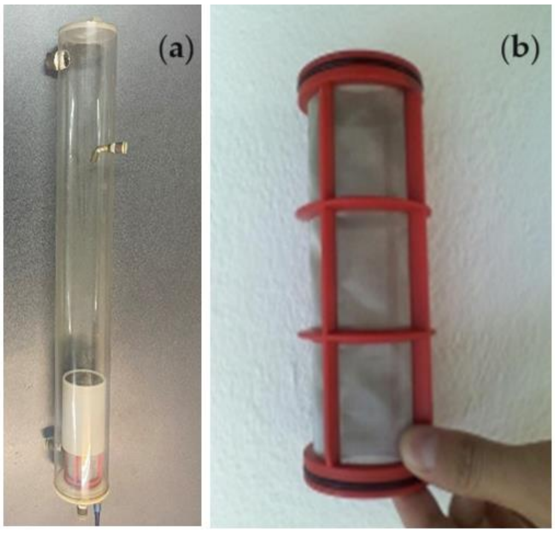

Figure S2. The p–nitrophenol reduction reaction column (a); and the detail of the emulsion (b).
